# Circulating miRNAs in pediatric obesity: a single-center exploratory study of their potential association with renal function

**DOI:** 10.3389/fmolb.2026.1851386

**Published:** 2026-06-10

**Authors:** Mariella Valenzise, Selene Francesca Anna Drago, Francesca Polito, Giuseppina Salzano, Irene Gasparo, Marieme Khouyyi, Laura Licitri, Anna Maria Mirabello, Silvia Romano, Valentina Mottola, Rosario Vitale, Malgorzata Wasniewska, Angela Alibrandi, M’Hammed Aguennouz, Vincenzo Macaione

**Affiliations:** 1 Regional Reference Center for Pediatric Obesity, Pediatric Unit “G. Martino”, Messina, Italy; 2 Department of Human Pathology of Adulthood and Childhood, University of Messina, Messina, Italy; 3 Department of Chemical, Biological, Pharmaceutical and Environmental Sciences, University of Messina, Messina, Italy; 4 Department of Clinical and Experimental Medicine, University of Messina, Messina, Italy; 5 Northwell Health, New York City, NY, United States

**Keywords:** biomarkers, circulating microRNA, kidney damage, molecular signature, pediatric obesity

## Abstract

**Background:**

Childhood obesity is a growing global public health concern, with increasing prevalence and significant long-term health implications. MicroRNAs (miRNAs), small non-coding RNAs, have emerged as stable, easily measurable, and tissue-specific biomarkers. However, data supporting their role as biomarkers of kidney involvement in pediatric patients with essential obesity remain limited. This study aims to evaluate the potential association between circulating miRNAs and renal function in this population.

**Materials and Methods:**

This is a single-center, observational, analytical, cross-sectional study. This study evaluated a cohort of pediatric patients with essential obesity followed at the “Regional Reference Center for Pediatric Obesity, Pediatric Unit of A.O.U. G. Martino of Messina.” Clinical and biochemical characteristics were assessed; serum samples were analyzed to evaluate circulating miRNA profiles.

**Results:**

In the analyzed samples, statistically significant correlations were observed between selected circulating miRNAs (miR-16-5p, miR-18a-5p, miR-126-3p, and let-7d) and markers of renal function.

**Conclusion:**

This preliminary study identified correlations between specific miRNAs and kidney function parameters, potentially representing molecular signals associated with subclinical variations in renal status in the context of obesity-related nephropathy.

## 1 Introduction

Childhood obesity represents a major public health challenge worldwide, with its prevalence continuing to increase in both developed and developing countries (Istituto Superiore di Sanità, 2021; Meldrum et al., 2017). The World Health Organization (WHO) defines obesity as an excessive and generalized accumulation of body fat that poses significant health risks and recognizes it as a global epidemic, coining the term “globesity” (Nittari et al., 2019). Recent data from the Childhood Obesity Surveillance Initiative (COSI) and the Italian OKkio alla SALUTE (Istituto Superiore di Sanità, 2024) program reveal that 20% of Italian children are overweight, while approximately 10% are obese. Severe obesity affects more than 2.6% of the pediatric population and displays a marked geographical gradient, with prevalence increasing progressively from northern to southern regions. Obesity, beginning in childhood, often persists into adulthood and is associated with an increased risk of cardiovascular (Sommer and Twig, 2018; Umer et al., 2017; Valerio et al., 2024), metabolic (Reilly and Kelly, 2011), hepatic (Mann J. et al., 2018; Marzuillo, 2014), respiratory (Forno et al., 2018), orthopedic (Maggio et al., 2014), and renal complications (Di Ludovico et al., 2025); several of these complications may already manifest during the first decades of life. Growing evidence suggests that obesity is associated with an increased risk of kidney complications in children (Di Ludovico et al., 2025; Forcina et al., 2024). A landmark large-scale cohort study demonstrated a robust and independent association between elevated body mass index (BMI) and the subsequent development of chronic kidney disease (CKD) and end-stage renal disease (ESRD), even after adjustment for traditional risk factors (Hsu et al., 2006). More recent observational data further confirm that overweight and obese individuals have significantly increased odds of CKD and ESRD (Ghazy et al., 2024; Kanbay et al., 2023), highlighting how obesity-related nephropathy often progresses insidiously and remains asymptomatic in its early stages.

Despite this evidence, current diagnostic tools for renal impairment in obese children still have limited sensitivity and specificity, leading to delays in diagnosis and treatment that may compromise patients’ clinical outcomes (Ding and Mak, 2015; Goknar et al., 2015). Serum creatinine, the most commonly used renal marker, is a late and non-specific indicator influenced by factors such as age, sex, muscle mass, and nutritional status (Sandilands et al., 2013). In recent years, several new biomarkers have been evaluated and validated. Among these, cystatin C, a low-molecular-weight serum protein, has proved to be a more reliable indicator of kidney function as it is independent of sex and muscle mass (Herget-Rosenthal et al., 2007). Currently, novel biomarkers (Ennulat and Adler, 2015) such as neutrophil gelatinase-associated lipocalin (NGAL) and kidney injury molecule-1 (KIM-1) have shown promise in identifying early tubular alterations; however, further validation is required, particularly in pediatric populations (Polidori et al., 2020).

In recent years, microRNAs (miRNAs) have emerged as a new class of potential non-invasive biomarkers. These small non-coding RNAs regulate post-transcriptional gene expression and play key roles in renal cell proliferation, differentiation, and apoptosis (Bartel, 2004; Bartel, 2018; Chandrasekaran et al., 2012; Mukhadi et al., 2015; Pu et al., 2019). miRNAs are detectable in a wide range of biological fluids, including serum and plasma. They exhibit remarkable stability, largely attributable to their encapsulation within extracellular vesicles such as exosomes, which protect them from enzymatic degradation (Coenen-Stass et al., 2019; Weber et al., 2010). Many miRNAs exhibit organ-specific expression patterns, making them promising biomarkers for the early diagnosis of a wide range of diseases (Ata et al., 2025; Reid et al., 2011). In adults, altered miRNA expression profiles have been consistently associated with obesity-related kidney injury, suggesting their potential role as mediators of disease pathogenesis and early detection biomarkers. miR-21 is consistently upregulated in individuals with obesity and promotes renal fibrosis by acting on anti-fibrotic pathways, while the miR-29 family exhibits anti-fibrotic properties and is downregulated in obesity (Caus et al., 2021; Earle et al., 2022; Metzinger-Le Meuth and Metzinger, 2023).

However, despite the growing body of evidence regarding miRNAs in the adult populations, data on their expression profiles, functional significance, and potential utility as non-invasive biomarkers in children and adolescents with obesity remain limited. This gap in knowledge highlights the urgent need for further research to better understand the early molecular changes associated with pediatric obesity and assess the role of miRNAs as tools for the diagnosis of obesity-related complications. In line with the study’s focus, this work aims to explore the association between circulating miRNAs and renal function in pediatric patients with obesity, evaluating their role as potential molecular signals associated with subclinical variation in renal function, and their possible utility in supporting earlier diagnosis and timely interventions to prevent progression toward chronic kidney disease (CKD).

## 2 Materials and methods

A single-center, observational, analytical, cross-sectional study was conducted using prospectively collected data from a pediatric population with essential obesity followed at the Regional Reference Center for Pediatric Obesity, Pediatric Unit of A.O.U. G. Martino, Messina.

Patients aged 5–16 years with essential obesity, of both sexes and without racial restrictions, were selected. Obesity was defined by a body mass index (BMI), calculated as weight (kg)/height (m^2^) ≥ 97th percentile according to Endocrine Society Clinical Practice Guideline (Styne et al., 2017).

Moreover, additional data were collected regarding patient management according to the PDTA (Integrated Care Pathway), including the following:

- Clinical–auxological evaluations include detailed medical history and measurement of weight, height, BMI, waist and hip circumference, and blood pressure.
- Laboratory parameters included the following: fasting glucose (mg/dL), fasting insulin (mU/L), HbA1c (%), total cholesterol (mg/dL), HDL cholesterol (HDL-C) (mg/dL), LDL cholesterol (LDL-C) (mg/dL), triglycerides (mg/dL), triglycerides/HDL ratio, HOMA-IR, AST (GOT, U/L), ALT (GPT, U/L), alkaline phosphatase, blood urea nitrogen (BUN) (mg/dL), serum creatinine (mg/dL), uric acid (mg/dL), Na^+^ (mmol/L), K^+^ (mmol/L), and hemoglobin (Hb) (g/dL).
- Other related assessments are performed in an outpatient setting, day service, or during hospitalization.

Therefore, participation in the study did not involve any additional risk for the patients. The overweight index was calculated as (actual–ideal weight) 100/ideal weight (actual–ideal weight) × 100/ideal weight, with values ≥40% indicating moderate-to-severe obesity. Hip and waist circumferences and their ratios were measured. Lipid abnormalities were defined as follows: total cholesterol ≥200 mg/dL, LDL-C ≥ 130 mg/dL, HDL-C ≤ 40 mg/dL, triglycerides ≥100 mg/dL (<10 years) or ≥130 mg/dL (>10 years), and an HDL-to-triglyceride ratio ≥2.2, indicating a pro-atherogenic profile. Insulin resistance was assessed by HOMA-IR > 3.16 and fasting insulin ≥15 mU/L or OGTT 120-min insulin ≥75 mU/L. Pre-diabetes was defined as fasting glucose 100–126 mg/dL or OGTT ≥140 mg/dL; type 2 diabetes was defined as fasting glucose >126 mg/dL or OGTT ≥200 mg/dL. Transaminase elevation was defined as AST/ALT ≥ 50 U/L. The glomerular filtration rate (eGFR) was estimated using the Schwartz “bedside” formula, which is the most commonly used method in pediatric populations (Schwartz et al., 2009). eGFR values ≥90 mL/min/1.73 m^2^ were considered normal, while eGFR values <90 mL/min/1.73 m^2^ were considered reduced.

During the visit, serum samples were collected for miRNA extraction. Patients were excluded from the study if they were older than 16 years or if they had syndromic obesity or obesity secondary to metabolic disorders. Individuals with clinical and/or laboratory evidence of pre-existing renal dysfunction, including primary, genetic, or secondary nephrological diseases, were also excluded. Patients taking medications that could affect renal function were not included in the study. The study was authorized by the Ethics Committee of the AOU G. Martino Messina Hospital (study code 1972; protocol num. 15918).

### 2.1 Selection of miRNAs

In order to select a panel of specific miRNAs as possible biomarkers of kidney damage in children with obesity, we performed a comprehensive search on PubMed and ScienceDirect using the keywords “miRNA,” “biomarkers,” “plasma,” “kidney,” and “obesity.” We thus selected, from three articles (Caus et al., 2021; Earle et al., 2022; Metzinger-Le Meuth and Metzinger, 2023), some miRNAs as potential biomarkers of obesity-related nephropathy in our patient cohort.

### 2.2 Sample processing and miRNA extraction

Blood samples were collected from 30 pediatric patients with essential obesity and 10 normal-weight controls. miRNA analysis has been performed on serum samples from 30 cases and all controls. Blood was collected in BD Vacutainer tubes with gel separators to avoid hemolysis.

Serum was obtained by centrifugation at 3,500 rpm for 15 min at 4 °C, followed by a second centrifugation to remove residual cells. Aliquots were stored at −80 °C until analysis. Total RNA was extracted from 800 μL of serum using the QIAGEN miRNeasy Kit, according to the manufacturer’s protocol for small RNAs. RNA quantification was assessed using a Bioanalyzer tool (Agilent Technologies, Santa Clara, CA, United States). Circulating miRNA was reverse-transcribed into cDNA using the TaqMan MicroRNA Reverse Transcriptase Kit (Thermo Fisher Scientific). miRNA expression was quantified by RT-PCR (AB-7300 system) using 2 μL of cDNA per reaction with specific TaqMan assays. All reactions, including pooled miRNA controls, no-template, and RT controls, were performed in triplicate (Polito et al., 2020). RNU6 was used for normalization. Relative expression was calculated using the 2^−ΔΔCt^ method, and the results are reported as fold change (log_2_RQ) relative to normal controls. Furthermore, pathway analysis was performed using miRPathv4 (http://62.217.122.229:3838/app/miRPathv4) using the KEGG PATHWAY database with miRTarBase 2022 as the target (Tastsoglou et al., 2023; Kanehisa and Goto, 2000; Kanehisa et al., 2023). This analysis confirmed that these miRNAs are associated with multiple signaling pathways implicated in renal pathophysiology, including the mTOR, Fox0, p53, PI3K–AKT, and AGE–RAGE pathways.

### 2.3 Statistical analysis

Data were summarized using a descriptive exploratory approach. Continuous variables are presented as the means ± standard deviations (SD) and ranges, whereas categorical variables are expressed as absolute frequencies and percentages. Relative miRNA expression levels were assessed using the 2^−ΔΔCt^ method, with fold changes calculated relative to normal controls and reported as log_2_RQ values. To explore potential associations between miRNA expression and clinical or biochemical parameters, correlation analyses were performed using Pearson’s or Spearman’s tests, depending on whether the variables followed a normal distribution. Statistical significance was defined as a *p*-value <0.05. All analyses were conducted using SPSS for Windows, version 23.0.

## 3 Results

**FIGURE 1:**
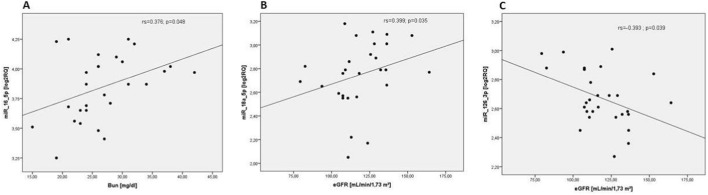
**(A)** Correlation between miR-16-5p expression and BUN. **(B)** Correlation between miR-18a-5p expression and eGFR. **(C)** Correlation between miR-126-3p expression and eGFR.

A total of 30 pediatric patients with essential obesity were included in the analysis. Of the 30 patients, 18 (60%) were female and 12 (40%) were male, with an age (years) of 11.25 ± 2.55 SD (Table 1). Among the 10 subjects in the control group, 6 (60%) were female and 4 (40%) were male, with a mean age of 11.50 ± 0.50 years (SD). The characteristics of the control group are reported in the Supplementary Material (Table 1). In addition, within the study group, 11 (36.7%) had mild obesity, while 19 (63.3%) had severe obesity, indicating that the majority of the evaluated patients presented with a more severe form of the condition. The levels of pubertal development were assessed according to Tanner stages (Marshall and Tanner, 1969; Marshall and Tanner, 1970), showing the following distribution among the patients: pre-pubertal, 33.3% (10 patients); puberty in progress, 36.7% (11 patients); and puberty completed, 30% (9 patients). The selected miRNAs showed higher expression levels in our cohort compared to controls, as indicated by log_2_RQ values (Table 2). All patients had serum creatinine, BUN (blood urea nitrogen), and uric acid values within the normal range for their age, with the following average values: creatinine (mg/dL) 0.5 ± 0.12 SD and BUN (mg/dL) 27 ± 6.1 SD. The eGFR assessment revealed that the children in our study had an average eGFR of 118.7 mL/min/1.73 m^2^ (±18 SD). Due to missing creatinine measurements in 2 children (6.7%), eGFR could not be calculated in these cases. Of the remaining 28, 2 (7.1%) had reduced eGFR (<90 mL/min/1.73 m^2^), while 26 (92.9%) had eGFR values >90 mL/min/1.73 m^2^. Five of these children (19.2%) had eGFR values above the upper limits of the normal range for their age. The cardio-metabolic risk of the patients was assessed through metabolism profiling and showed that 3 (10%) patients had hypercholesterolaemia, 6.7% had elevated LDL-C values, and 17.9% had decreased HDL-C levels. Impaired fasting glucose was observed in 40% of subjects. Among the 21 patients who underwent an oral glucose tolerance test, 15 (71.4%) exhibited insulin resistance, while 3 (15%) showed impaired glucose tolerance. Ultrasound examination of the liver was performed in 24 patients (80%). Of these, 15 (62.5%) showed a normal ultrasound appearance, while 9 (37.5%) presented with hepatic steatosis. To identify the miRNAs most closely associated with the disease, correlation analyses were conducted between differentially expressed miRNAs (showed higher expression levels) and various biochemical parameters. The study revealed several significant correlations, summarized as follows:

- miR_16_5p positively correlated with BUN values (rs = 0.376; *p*-value 0.048) (Figure 1A);
- miR_18a _5p positively correlated with eGFR values (rs = 0.399; *p*-value 0.035) (Figure 1B);
- miR_126_3p inversely correlated with eGFR values (rs = 0.393; *p*-value 0.039) (Figure 1C) and also positively correlated with creatinine values (rs = 0.451; *p*-value 0.016); and
- Let_7d positively correlated with glomerular hypofiltration (eGFR<90) (rs = 0.379; *p*-value 0.047); however, this analysis is based on a limited number of patients and should be considered preliminary.

**TABLE 1:** Descriptive analysis of clinical and biochemical parameters of the study population.

Variable (N = 30)	Average	Standard deviation	Minimum	Maximum
Clinical parameter				
Age (years)	11.3	2.6	6.5	15.4
SBP (mmHg)	116.0	11.1	98.0	150.0
DBP (mmHg)	68.7	9.7	51.0	90.0
Weight (kg)	70.9	23.5	43.0	154.0
Ideal weight/height (%)	161.0	24.9	126.2	225.7
WtI (kg)	42.2	10.2	24.7	55.8
Height (cm)	149.5	14.2	123.1	181.4
BMI (kg/m^2^)	31.1	5.6	23.8	46.8
Waist circumference (cm)	92.4	8.7	78.0	114.0
Hip circumference (cm)	95.0	23.9	74.0	125.5
Waist-to-hip ratio	0.93	0.1	0.8	1.1
Waist-to-height ratio	0.62	0.1	0.5	0.8
Biochemical parameters				
Fasting glucose (mg/dL)	99.7	9.9	84.0	125.0
OGTT glucose (mg/dL) (N = 21)	128.5	39.6	85.0	284.0
Fasting insulin (mU/L)	23.0	12.2	6.9	65.3
OGTT insulin (mU/L) (N = 21)	125.1	94.6	11.7	454.0
HbA1c (%)	5.5	0.2	5.1	5.9
Total cholesterol (mg/dL)	161.8	27.7	118.0	236.0
HDL-C (mg/dL)	46.2	6.8	31.0	59.0
LDL-C (mg/dL)	94.8	21.3	62.0	149.0
Triglycerides (mg/dL)	83.9	38.9	33.0	206.0
TG/HDL ratio	1.8	0.9	0.6	4.9
HOMA-IR	5.7	3.5	1.6	18.7
AST (U/L)	28.3	10.7	14.0	64.0
ALT (U/L)	27.0	17.5	11.0	98.0
ALP (U/L)	208.4	82.3	91.0	381.0
BUN (mg/dL)	27.0	6.2	15.0	42.0
Serum creatinine (mg/dL) (N = 28)	0.53	0.13	0.3	0.8
eGFR (mL/min/1.73 m^2^) (N = 28)	118.7	18.7	79.4	164.2
Uric acid (mg/dL)	5.1	1.4	3.0	8.4
Na^+^ (mmol/L)	138.8	1.8	135.0	142.0
K^+^ (mmol/L)	4.4	0.3	3.0	5.2
Hb (g/dL)	13.0	1.1	10.6	15.3

SBP, systolic blood pressure; DBP, diastolic blood pressure; WtI, ideal body weight; BMI, body mass index; OGTT-G, oral glucose tolerance test glucose; OGTT-I, oral glucose tolerance test insulin; HbA1c, glycated hemoglobin; HDL-C, high-density lipoprotein cholesterol; LDL-C, low-density lipoprotein cholesterol; TG/HDL ratio, triglyceride-to-HDL cholesterol ratio; HOMA-IR, homeostasis model assessment of insulin resistance; AST, aspartate aminotransferase; ALT, alanine aminotransferase; ALP, alkaline phosphatase; BUN, blood urea nitrogen; eGFR, estimated glomerular filtration rate; Na^+^, serum sodium; K^+^, serum potassium; Hb, hemoglobin.

**TABLE 2:** MiRNA descriptive analysis.

miRNAs	Average	Standard deviation	Minimum	Maximum
miR_16_5p	3.82	0.29	3.21	4.25
miR_34a_5p	3.54	0.45	2.87	4.13
miR_103a_3p	3.05	0.38	2.54	4.05
miR_107	2.99	0.37	2.51	4.05
miR_124_3p	2.80	0.23	2.05	3.18
miR_33a	2.99	0.09	2.79	3.14
miR_27a	2.90	0.37	2.05	4.01
miR_18a_5p	2.73	0.29	2.05	3.18
miR_146_5p	2.85	0.29	2.48	4.02
let_7d	2.50	0.28	1.89	2.98
miR_15b_5p	2.54	0.44	1.05	3.02
miR_130b_3p	2.78	0.18	2.45	3.28
miR_142_5p	2.79	0.29	2.47	4.01
miR_126_3p	2.66	0.19	2.25	3.01
miR_152_3p	2.77	0.46	1.95	4.01
miR_423_3p	2.71	0.37	1.90	4.11
miR_425_3p	2.71	0.23	2.02	3.11
miR_523_3p	2.75	0.22	2.09	3.06
miR_766_3p	2.72	0.24	1.99	3.25

**FIGURE 2:**
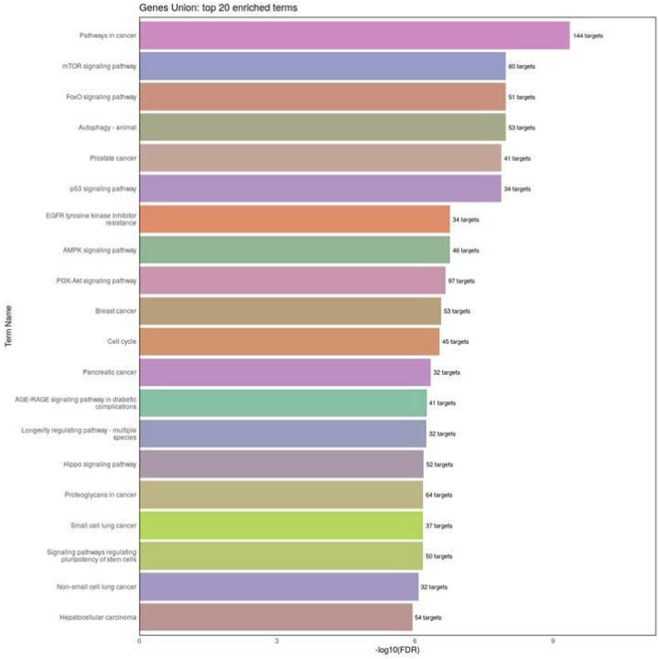
Gene enrichment analysis using the KEGG PATHWAY database identified mTOR, Fox0, p53, PI3K–AKT, and AGE–RAGE signaling pathways among the most significant pathways associated with the miRNAs studied.

A detailed analysis was conducted of the potential involvement of miRNAs in metabolic pathways related to renal function, using miRPath v4 (KEGG PATHWAY Database), revealing their association with various molecular signaling networks that may influence renal physiology (Figure 2).

Furthermore, additional correlations with metabolic and clinical parameters were observed, including (Table 3)

- miR_103a_3p correlated directly with OGTT-G (rs = 0.457; *p*-value <0.05);
- miR_130b_3p correlated directly with OGTT-I (rs = 0.455; *p*-value <0.05); and
- miR_27 a correlated directly with LDL-C (rs = 0.397; *p*-value <0.05) and inversely with HDL-C (rs = −0.463; *p*-value <0.05).

**TABLE 3:** Statistical correlations between miRNAs and metabolic/clinical parameters.

miRNAs	Parameter	Correlation coefficient (Spearman’s)	*p*-value
miR_103a_3p	OGTT-G	0.457	0.043
miR_130b_3p	OGTT-I	0.455	0.038
miR_27a	HDL-C	−0.463	0.013
miR_27a	LDL-C	0.397	0.036
miR_33a	AST	0.414	0.026
miR_27a	AST	0.441	0.017
miR_27a	OGTT-I	−0.579	0.006
miR_27a	Degree of obesity	0.417	0.025

OGTT-G, oral glucose tolerance test glucose; OGTT-I, oral glucose tolerance test insulin; HDL-C, high-density lipoprotein cholesterol; LDL-C, low-density lipoprotein cholesterol; AST, aspartate aminotransferase.

## 4 Discussion

Obesity, notably among children but progressively among adults, represents a significant public health concern, with global prevalence steadily increasing. Childhood obesity not only predisposes individuals to the persistence of excess adiposity into adulthood but also significantly increases the risk of associated complications, including cardiovascular, metabolic, hepatic, and renal disorders (many of which begin to manifest early in life) (Sawyer et al., 2023). The onset of obesity-related nephropathy is often insidious and asymptomatic, while conventional clinical diagnostic tests (e.g., serum creatinine and estimated glomerular filtration rate) have limited sensitivity for the early detection of renal damage and for accurately assessing the degree of renal functional impairment. One of the major consequences is diagnostic delay, which may result in missed opportunities for timely intervention and an increased likelihood of progression to ESRD (Medyńska et al., 2024). Prompt diagnosis of CKD is essential for reducing both the clinical impact and the associated long-term healthcare costs. Currently, no candidate biomarker meets all the criteria for an ideal marker, including ease of collection, stability, reliability in the presence of interfering factors, tissue- or organ-specificity, and a clear correlation between changes in expression and disease risk or prognosis. Increasing evidence supports the involvement of miRNAs in various processes, including the different stages of renal impairment (Earle et al., 2022; Franczyk et al., 2022). A more comprehensive analysis of expression patterns could provide further information on sensitivity and specificity, thereby reinforcing their potential utility as diagnostic and/or prognostic tools. Given the limited research in pediatric cohorts and the current gaps in literature, this study aimed to conduct a preliminary investigation of miRNA expression levels in pediatric patients with essential obesity. Various studies identified the involvement of specific miRNAs, including miR-146a (NF-κB signaling pathway), miR-103a-3p (SNRK pathway) (Lu et al., 2019; Mann et al., 2017; Mann M. et al., 2018; Peters et al., 2020), and miR-130b and miR-34a-5p (SIRT1 gene target, SIRT/TGF-β signaling pathway) (Tian et al., 2016; Wang et al., 2021), highlighting their potential role in the onset and/or progression and pathogenesis of obesity-related kidney disease in adults.

Our exploratory analysis revealed correlations between specific miRNAs and renal function parameters in our pediatric cohort. In detail, the analysis revealed a correlation between miR-16-5p and blood urea nitrogen. Previous literature has repeatedly identified this microRNA in the context of kidney injury, where its expression is thought to reflect kidney function, cellular stress, and damage (Chen et al., 2016). miR-18a-5p, together with miR-16-5p, has been identified as part of injury-responsive microRNA signatures in renal transplant and kidney injury studies. It is considered that this manifestation reflects adaptive or reparative processes rather than direct tissue damage, which could explain the observed positive association with preserved renal filtration capacity (higher eGFR) (Connor et al., 2020). In addition, miR-126-3p showed an inverse correlation with eGFR and a direct correlation with serum creatinine values, suggesting a possible association with renal function; however, these results should be interpreted with caution, given the exploratory nature of the analysis. These findings appear to be consistent with reports in the literature on adult CKD populations, in which reduced circulating levels of miR-126 have been associated with lower eGFR and more advanced stages of kidney disease (Fourdinier et al., 2019; Motshwari et al., 2023). Comparisons should be made with caution given the differences between the study populations and the study designs. Additionally, although Let-7d appeared to correlate with glomerular hypofiltration, this analysis should be interpreted with caution due to the very small number of affected patients. Nevertheless, it may be consistent with previous evidence implicating members of the Let-7 family in mechanisms of renal fibrosis and chronic kidney dysfunction (Lv et al., 2018). Furthermore, miR-18a-5p showed a direct correlation with eGFR, suggesting its potential role as a novel marker of glomerular involvement; however, this association warrants further validation in larger cohorts. Only a limited number of studies have examined this miRNA, and available evidence suggests a possible association with renal hyperfiltration in the early stages of obesity (Caus et al., 2021). Furthermore, our study showed that serum creatinine and BUN levels were within the normal range in all children, confirming that conventional biomarkers are relatively late and non-specific indicators of kidney involvement and may not to reliably detect early signs of impairment (Nassirpour et al., 2016). We also aimed to evaluate correlations with clinical and metabolic parameters, identifying associations between specific miRNAs and measures such as OGTT-G, OGTT-I, lipid profile, and degree of obesity. Several studies have shown that the profiles of circulating microRNAs are significantly altered in obese children compared with normal-weight control subjects and that these alterations are associated with anthropometric parameters (BMI, obesity, and waist circumference) or metabolic indicators (insulin resistance, lipid levels, and inflammation), as well as with adiposity phenotypes already evident in the early stages of life (Prats-Puig et al., 2013; Santos et al., 2023). In particular, the study by Prats-Puig et al. (2013) demonstrated that miR-486-5p, miR-486-3p, miR-142-3p, miR-130b, and miR-423-5p were upregulated in children with obesity, suggesting that specific miRNAs may reflect the presence of adiposity and metabolic dysfunction. A systematic review identified differentially expressed miRNAs in childhood obesity consistently altered and associated with obesity or related conditions, including insulin resistance and non-alcoholic fatty liver disease (NAFLD) (Oses et al., 2019). These observations reinforce the concept that miRNA dysregulation contributes to obesity-related complications through multi-organ involvement and modulation of key molecular pathways. Gene union enrichment analysis identified several enriched KEGG pathways among miRNA target genes (Figure 2), including mTOR, FoxO, PI3K–AKT, and p53 signaling. These pathways are primarily involved in metabolic regulation, cellular growth, and stress response mechanisms, supporting a potential role of the analyzed miRNAs in biological processes relevant to renal function. As key regulators of lipid and glucose metabolism, miRNAs modulate insulin signaling, adipogenesis, lipid homeostasis, and energy balance through their ability to simultaneously regulate multiple genes and molecular networks (Deiuliis, 2016; Iacomino and Siani, 2017). In particular, adipose tissue is now recognized as an endocrine organ that secretes miRNAs, which may act as novel adipokines, thereby influencing inflammation, insulin resistance, and systemic metabolic regulation (Heyn et al., 2020; Liu et al., 2024). Moreover, specific miRNAs have been reported to regulate insulin sensitivity, glucose metabolism, and related signaling pathways (Trajkovski et al., 2011). Furthermore, several miRNAs are involved in the regulation of lipid metabolism and adipocyte differentiation, linking their dysregulation to alterations in BMI and lipid profiles characteristic of obesity (Arner and Kulyté, 2015). These results support the hypothesis that miRNA expression profiles mirror systemic metabolic alterations, influencing auxological measures and key metabolic pathways. They may represent integrative biomarkers reflecting the complex interplay between adiposity, metabolic dysfunction, and organ-specific function. It has been suggested that the dysregulation of miRNA expression profiles in biological fluids may, over time, be associated with alterations in renal function during childhood and adolescence (Garmaa et al., 2024; Motshwari et al., 2023). The variability in miRNAs observed across studies may reflect differences in population, age, degree of obesity, and analytical methodologies. Overall, the evidence suggests that circulating miRNA profiles are not merely passive indicators of obesity but may also be involved in the regulation of key metabolic processes. In this context, the simultaneous measurement of circulating miRNAs may represent a valuable diagnostic tool for identifying risk profiles associated with progression toward metabolic complications. However, given the limited evidence available, further studies are needed to better characterize these findings, explore their potential association with renal function, and evaluate their possible application within standardized diagnostic approaches for pediatric obesity-related nephropathy.

## 5 Conclusion

miRNAs are increasingly recognized as key regulators of fundamental biological processes. Although their role in obesity-related nephropathy and its associated complications has not yet been fully elucidated, evidence in this field is rapidly expanding. Results from our preliminary pediatric study seem to align with observations reported in the literature; circulating miRNA profiles may reflect non-random patterns, potentially representing molecular signals associated with subclinical variations in renal function. The growing understanding of miRNAs in modern medicine underscores their transformative potential: they could facilitate earlier diagnosis, enable refined risk stratification, and support the development of more targeted interventions. Furthermore, the identification of specific miRNA signatures may support precision medicine approaches, providing opportunities for individualized prediction, prevention, and therapeutic strategies in the management of obesity-related complications. However, further studies are needed to confirm these results and clarify miRNAs’ potential role as early markers in obesity-related nephropathy.

## 6 Limitations and future perspectives

The main limitation of this study is the relatively small sample size. As a future direction, we plan to expand our research by enrolling a larger cohort of children with obesity, in whom miRNA expression levels will be assessed not only in serum but also in urine samples. Furthermore, given the exploratory nature of the analysis and the limited sample size, some observed associations are borderline and may not remain significant after correction for multiple testing; therefore, these findings should be interpreted with caution. Larger cohorts will be essential to confirm these results and to enable more robust and reliable statistical analyses. In addition, no multivariable analyses were performed due to the limited sample size; thus, potential confounding factors may have influenced the observed associations. Future investigations will also aim to correlate miRNA levels with additional renal function parameters, such as microalbuminuria and renal ultrasound, and to perform stratifications based on age and pubertal stage. Another limitation of this study is the use of RNU6 as a reference gene as its stability in serum was not specifically validated in our cohort and may vary according to the biological matrix and experimental conditions. Ultimately, the goal is to improve diagnostic strategies by identifying miRNAs with high specificity that may serve as reliable biomarkers for obesity-related nephropathy.

## Data Availability

The raw data supporting the conclusions of this article will be made available by the authors, without undue reservation.
