# Supplementary material for: Circulating miRNAs in pediatric obesity: a single-center exploratory study of their potential association with renal function
Source: Front Mol Biosci. 2026 Jun 10;13:1851386. doi: 10.3389/fmolb.2026.1851386 (PMC13290197; doi:10.3389/fmolb.2026.1851386)
Supplement: Supplementary file 1 [file Table1.docx]

**Supplementary Material. Table 1. Descriptive Analysis of Clinical and Biochemical Characteristics of Control Group.**

| **Variables (N=10)** | **Average** | **Standard Deviation** | **Minimum** | **Maximum** |
| --- | --- | --- | --- | --- |
| **Clinical Parameters** |  |  |  |  |
| Age (years) | 11.5 | 0.5 | 11.0 | 12.0 |
| SBP (mmHg) | 112.11 | 7.23 | 104.88 | 119.34 |
| DBP (mmHg) | 64.41 | 7.32 | 57.09 | 71.73 |
| Weight (kg) | 43.82 | 13.42 | 30.40 | 57.24 |
| Height (cm) | 148.31 | 12.88 | 135.43 | 161.19 |
| BMI (kg/m²) | 17.35 | 4.72 | 12.63 | 22.07 |
| Waist Circumference (cm) | 66.61 | 7.56 | 59.05 | 74.17 |
| Hip Circumference (cm) | 77.13 | 18.21 | 58.92 | 95.34 |
| Waist-to-hip ratio | 0.75 | 0.06 | 0.69 | 0.81 |
| Waist-to-height ratio | 0.44 | 0.15 | 0.29 | 0.59 |
| **Biochemical parameters** |  |  |  |  |
| Fasting Glucose (mg/dL) | 88.46 | 6.45 | 82.01 | 94.91 |
| OGTT Glucose (mg/dL) | 110.23 | 10.26 | 99.97 | 120.49 |
| Fasting Insulin (mU/L) | 8.03 | 2.36 | 5.67 | 10.39 |
| OGTT Insulin (mU/L) | 74.54 | 6.35 | 68.19 | 80.89 |
| HbA1c (%) | 4.80 | 0.20 | 4.60 | 5.00 |
| Total Cholesterol (mg/dL) | 148.23 | 22.38 | 125.85 | 170.61 |
| HDL-C (mg/dL) | 44.56 | 5.47 | 39.09 | 50.03 |
| LDL-C (mg/dL) | 82.65 | 16.32 | 66.33 | 98.97 |
| Triglycerides (mg/dL) | 66.41 | 18.32 | 48.09 | 84.73 |
| TG/HDL Ratio | 1.53 | 0.54 | 0.99 | 2.07 |
| HOMA-IR | 1.70 | 0.32 | 1.38 | 2.02 |
| AST (U/L) | 26.54 | 8.62 | 17.92 | 35.16 |
| ALT (U/L) | 25.47 | 7.89 | 17.58 | 33.36 |
| ALP (U/L) | 203.27 | 76.36 | 126.91 | 279.63 |
| BUN (mg/dL) | 10.88 | 2.25 | 8.63 | 13.13 |
| Serum Creatinine (mg/dL) | 0.48 | 0.16 | 0.32 | 0.64 |
| eGFR (mL/min/1.73 m²) | 98.52 | 16.54 | 81.98 | 115.06 |
| Uric Acid (mg/dL) | 3.86 | 1.66 | 2.20 | 5.52 |
| Na⁺ (mmol/L) | 138.28 | 2.15 | 136.13 | 140.43 |
| K⁺ (mmol/L) | 3.92 | 0.75 | 3.17 | 4.67 |

Systolic blood pressure (SBP). Diastolic blood pressure (DBP). ideal body weight (WtI). body mass index (BMI). oral glucose tolerance test glucose (OGTT-G). oral glucose tolerance test insulin (OGTT-Insulin). glycated hemoglobin (HbA1c). high-density lipoprotein cholesterol (HDL-C). low-density lipoprotein cholesterol (LDL-C). triglyceride-to-HDL cholesterol ratio (TG/HDL ratio). homeostasis model assessment of insulin resistance (HOMA-IR). aspartate aminotransferase (AST). alanine aminotransferase (ALT). alkaline phosphatase (ALP). blood urea nitrogen (BUN). estimated glomerular filtration rate (eGFR). serum sodium (Na⁺). serum potassium (K⁺).
